# Cilgavimab/Tixagevimab as alternative therapeutic approach for BA.2 infections

**DOI:** 10.3389/fmed.2022.1005589

**Published:** 2022-09-29

**Authors:** Stefanie Dichtl, Viktoria Zaderer, Viktoria Kozubowski, Hussam Abd El Halim, Eliott Lafon, Lukas Lanser, Günter Weiss, Cornelia Lass-Flörl, Doris Wilflingseder, Wilfried Posch

**Affiliations:** ^1^Institute of Hygiene and Medical Microbiology, Medical University of Innsbruck, Innsbruck, Austria; ^2^Department of Internal Medicine II, Medical University of Innsbruck, Innsbruck, Austria

**Keywords:** Tixagevimab/Cilgavimab, antibody therapies, COVID-19, 3D tissue cell cultures, lung model, SARS-CoV-2, Omicron BA.1 and BA.2

## Abstract

**Objectives:**

The identification of the SARS-CoV-2 Omicron variants BA.1 and BA.2 immediately raised concerns about the efficacy of currently used monoclonal antibody therapies. Here, we analyzed the activity of Sotrovimab and Regdanvimab, which are used in clinics for treatment of moderate to severe SARS-CoV-2 infections, and Cilgavimab/Tixagevimab, which are approved for prophylactic use, against BA.1 and BA.2 in a 3D model of primary human bronchial epithelial cells.

**Methods:**

Primary human airway epithelia (HAE) cells in a 3D tissue model were infected with clinical isolates of SARS-CoV-2 Delta, BA.1 or BA.2. To mimic the therapeutic use of mAbs, we added Regdanvimab, Sotrovimab or Cilgavimab/Tixagevimab 6 h after infection. In order to mirror the prophylactic use of Cilgavimab/Tixagevimab, we added this compound 6 h prior to infection to the fully differentiated, pseudostratified epithelia cultured in air-liquid interphase (ALI).

**Results:**

We observed that Sotrovimab, but not Regdanvimab, is active against BA.1; however, both antibodies lose their efficacy against BA.2. In contrast, we found that BA.2 was sensitive to neutralization by the approved prophylactic administration and the therapeutic use, which is not yet permitted, of Cilgavimab/Tixagevimab.

**Conclusion:**

Importantly, while the use of Tixagevimab/Cilgavimab is effective in controlling BA.2 but not BA.1 infection, monoclonal antibodies (mAbs) with efficacy against BA.1 are ineffective to reduce BA.2 virus replication in a human lung model. Our data may have implications on the variant specific clinical use of monoclonal antibodies.

## Background

Novel SARS-CoV-2 variants of concern (VOC) rapidly emerge. BA.1 (B.1.1.529.1) and BA.2 (B.1.1.529.2), exert a higher transmission rate compared to former variants including Delta (B.1.617.2) as well as high numbers of mutations within the spike protein, including the receptor-binding domain (RBD) (1). BA.2, contains 8 unique spike mutations while lacking 13 spike mutations found in BA.1 (2). Many monoclonal antibodies (mAbs) in clinical use target the RBD and, therefore, many have an impaired or no activity against the Omicron variants in neutralization assays. Neutralization assays are usually performed in Vero cells that significantly differ from human primary airway cells (3). This in turn could affect receptor binding and cellular entry blockade by mAbs. Moreover, many neutralization studies use pseudoviruses that could be different in neutralization compared to clinical isolates using particular mAbs (4).

Therefore, we investigated the efficacy of Emergency Use Authorization (EUA) mAbs, which are in clinical use in Austria in April 2022. For the early treatment of mild to moderate infections in patients at risk for a severe course of the infection, the mAbs Regdanvimab (Regkirona; CT-P59) and Sotrovimab (Xevudy; S309) are in clinical use. For prophylaxis of infection in subjects with high risk for severe COVID-19, the antibody combination of Cilgavimab and Tixagevimab (Evusheld) has been approved for clinical use.

## Methods

### Ethics statement

Written informed consent was obtained from all donors of leftover bronchial specimens. The Ethics Committee of the Medical University of Innsbruck (ECS1166/2020) approved the use of anonymized leftover specimens of patients with COVID-19 for scientific purposes.

### Cell culture

#### Human airway epithelia

NHBE cells are available in our laboratory and routinely cultured in ALI as described (5). Briefly, cells (from one healthy donor) from passage 2 were cultured until they reached 80% confluence. This early passage reduces donor specific differences and therefore, the data are highly reliable (6). The cells were seeded onto collagen-coated 0.33-cm^2^ porous (0.4-μm) polyester membrane inserts. Cultures were maintained in a humidified atmosphere with 5% CO_2_ at 37°C and then transferred to ALI culture. The epithelium was expanded and differentiated using airway media.

#### Vero/TMPRSS2

VeroE6/TMPRSS2 is an engineered VeroE6 cell line expressing high levels of TMPRSS2, which is highly susceptible to SARS-CoV-2 infection and used to expand SARS-CoV-2 viruses from repositories as well as patient isolates.

#### Viruses

Clinical specimens for SARS-CoV-2 Delta (B.1.617.2), Omicron BA.1 (B.1.1.529 BA.1) and BA.2 (B.1.1.529 BA.2) from COVID-19 positive swabs, sequenced by the Austrian Agency for Health and Food Safety, Vienna, Austria were propagated and used subsequently to infect cells.

#### Virus infection

NHBE cells were cultured in ALI for 21–33 days. 50 μg/ml Evusheld was added to the basolateral side 6 h before or after infection. The cells were infected with an MOI of 0.01 of clinical specimen of SARS-CoV-2 apically. Where indicated, 50 μg/ml Regdanvimab or Sotrovimab was added to the basolateral side 6 h after infection. The cells were harvested on day 3 postinfection (3 dpi).

#### Transepithelial electrical resistance measurement

TEER values were measured using an EVOM voltohmmeter. Measurements on cells in ALI culture were taken 3 dpi. For measurements, 100 μl medium was added to the apical side. Cells were allowed to equilibrate before TEER was measured.

#### Immunofluorescence staining and imaging

After infection, cells were fixed and intracellular staining using Intracellular Staining Permeabilization Wash Buffer. Antibodies to detect nuclei (Hoechst 33342), actin (phalloidin-Alexa647) and complement C3 (C3-FITC) were used. Intracellular SARS-CoV-2 was detected using Alexa594-labeled SARS-CoV-2 antibodies against S1 and N. The Operetta CLS System (PerkinElmer) was used to image the samples. Analysis were done using the Harmony software (Perkin Elmer). For quantification analyses, at least 1,000 cells per condition (randomly chose) were analyzed as indicated. For detection of SARS-CoV-2-positive cells all conditions were normalized to UI due to background fluorescence in the UI, Sotrovimab, Regdanvimab and Evusheld-treated samples.

#### Real-time RT-PCR for absolute quantification of SARS-CoV-2

SARS-CoV-2 RNA was extracted using FavorPrep Viral RNA Mini Kit, according to manufacturer’s instructions. Sequences specific to 2 distinct regions of the Nucleocapsid (N) gene, N1 and N2, and for the detection of a human housekeeping gene, Ribonuclease P, were used. Single target assays of all 3 targets were performed in combination with the Luna Universal Probe One-Step RT-qPCR Kit. For absolute quantification using the standard curve method, SARS-CoV-2 RNA was obtained as a PCR standard control from the National Institute for Biological Standards and Control UK. All runs were performed on a Bio-Rad CFX 96 instrument and analyzed by the Bio-Rad CFX Maestro 1.1 software.

#### Plaque assay

Plaque Assay was modified from Lafon et al. (7). VeroE6/TMPRSS2 cells were seeded and on the next day, supernatant from the HAE cells was serial diluted and incubated with VeroE6/TMPRSS2 cells for 1 h at 37°C. After incubation, the supernatant was removed and culture medium containing 1.5% carboxymethylcellulose was added. Cells were incubated for 3 days before plaque visualization and counting. For this, cells were washed, fixed for 1 h at room temperature and stained using 0.5% crystal violet solution.

#### Profiling of cytokines

IL6 secretion of HAE tissue models was detected by the human IL6 ELISA MAX Deluxe Set (Biolegend) according to the manufacturer’s instructions.

#### Statistical analysis

The significance of differences in the experimental data were determined using GraphPadPrism software. All data involving statistics are presented as mean ± SEM. Statistically significant differences were determined by one-way ANOVA with Tukey correction. All experiments were independently repeated at least 3 times.

## Results

**FIGURE 1:**
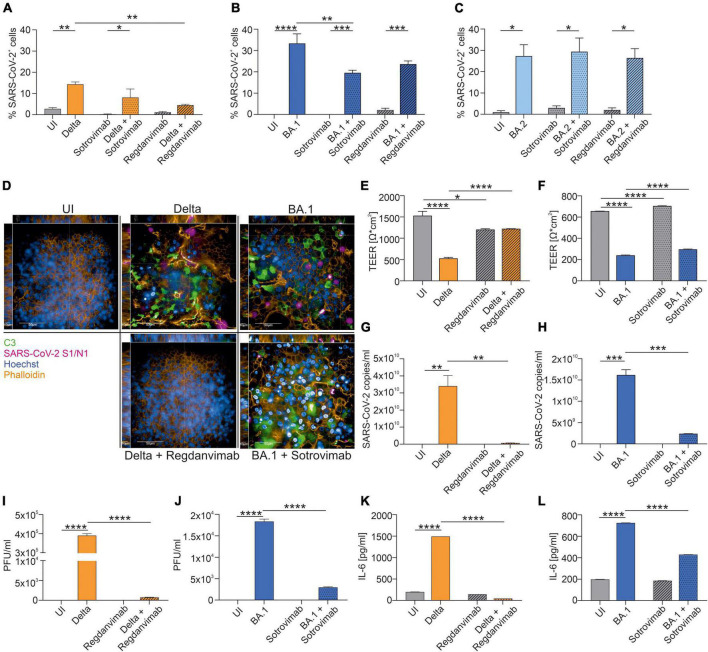
Neutralization efficacy of Sotrovimab and Regdanvimab against SARS-CoV-2 Delta, BA.1 or BA.2. After 72 h of infection, NHBE cells grown on transwell filters were analyzed by immunofluorescence with the Harmony Software and percentage of SARS-CoV-2-positive cells were determined for Delta- **(A)**, BA.1- **(B)** and BA.2-infected cells **(C)**. Background signals due to the analysis were detected in UI, Sotrovimab and Regdanvimab treated cells. **(D)** Representative pictures of XYZ stack are shown for uninfected (UI) cells and the conditions where Regdanvimab or Sotrovimab treatment resulted in a significant reduction of SARS-CoV-2-positive cells shown in **(A–C)**. Scale bars represent 50 and 10 μm as indicated. After 72 h of infection, TEER was measured from Delta- **(E)** or BA.1-infected HAE ± antibodies **(F)**. Viral RNA was analyzed in Delta- **(G)** and BA.1-infected cells ± antibodies **(H)**. Plaque Assays of supernatants from Delta- **(I)** and BA.1-infected cultures ± antibodies **(J)** were performed on Vero/TMPRSS2 cells. Concentration of IL-6 in the subnatants from Delta- **(K)** and BA.1-infected cultures ± antibodies **(L)** were measured by ELISA. Statistically significant differences were determined by one-way ANOVA with Tukey correction. All values are means ± SEM; *P < 0.05; **P < 0.01; ***P < 0.001; ****P < 0.0001. At least three independent experiments were performed.

We infected primary normal human bronchial epithelial (NHBE) cells in a 3D tissue model with clinical isolates of SARS-CoV-2 Delta, BA.1 or BA.2. To mimic the therapeutic use of mAbs, we added a physiological concentration of Regdanvimab or Sotrovimab 6 h after infection (8, 9). In order to mirror the prophylactic use of Cilgavimab/Tixagevimab, we added this compound 6 h prior to infection to the fully differentiated, pseudostratified epithelia cultured in air-liquid interphase (ALI). After 72 h of infection, the tissue models were fixed and stained for immunofluorescence analysis using antibodies against SARS-CoV-2 spike 1 (S1) and nucleocapsid (N) proteins to detect virus, Hoechst stain for nuclei, complement component C3 as a marker for innate immune activation and Phalloidin for detecting the F-actin within the cytoskeleton. C3 complement activation in SARS-CoV-2-infected primary human airway epithelia (HAE) cells was previously shown to initiate a highly inflammatory microenvironment, resulting in tissue damage (5, 10). We analyzed and quantified the percentage of SARS-CoV-2-positive cells in Delta- (Figure 1A), BA.1-(Figure 1B) and BA.2-infected cells (Figure 1C) in absence/presence of Regdanvimab and Sotrovimab. While in Delta-infected HAE cells only Regdanvimab treatment resulted in a significant reduction of SARS-CoV-2-positive cells (*P* = 0.002), in BA.1-exposed cells solely Sotrovimab significantly reduced the numbers of SARS-CoV-2-infected cells (*P* = 0.005). In BA.2-infected cells, both mAbs failed to ameliorate the infection (Supplementary Figure 1). Therefore, we continued to analyze Delta-infected cells treated with Regdanvimab and BA.1-infected cells with Sotrovimab in more detail, and representative XYZ analyses are depicted in Figure 1D. Here, we found that Regdanvimab treatment entirely protected from Delta virus infection while Sotrovimab treatment reduced BA.1 infection of HAE cells (Figure 1D). Moreover, C3 activation of Regdanvimab treated cells was comparable to uninfected cells, whereas Sotrovimab could not completely abolish this inflammatory signal (Figure 1D). These results were further underlined by measuring transepithelial electrical resistance (TEER), an indicator for tissue integrity. In Delta-infected cells the mean TEER value was 527 Ω/cm^2^, which was rescued with Regdanvimab treatment to 1,223 Ω/cm^2^ (Figure 1E). Sotrovimab treatment also resulted in a significant, less distinct, increase of the TEER values after BA.1 infection (Figure 1F). The treatment with the two mAbs also reduced the numbers of infective viral particles in Delta- (Figure 1G) and BA.1-infected cells (Figure 1H), respectively, which was further highlighted by reduced viral titers in mAb-treated groups analyzed via plaque assay (Figures 1I,J). Finally, we evaluated the potency of Regdanvimab and Sotrovimab to decrease the presence of pro-inflammatory cytokines like IL-6 during SARS-CoV-2 Delta or BA.1 infection. While Regdanvimab treatment in Delta-infected cells completely abrogated IL-6 production (Figure 1K), Sotrovimab significantly decreased IL-6 levels compared to BA.1-infected cells without added antibody (Figure 1L).

**FIGURE 2:**
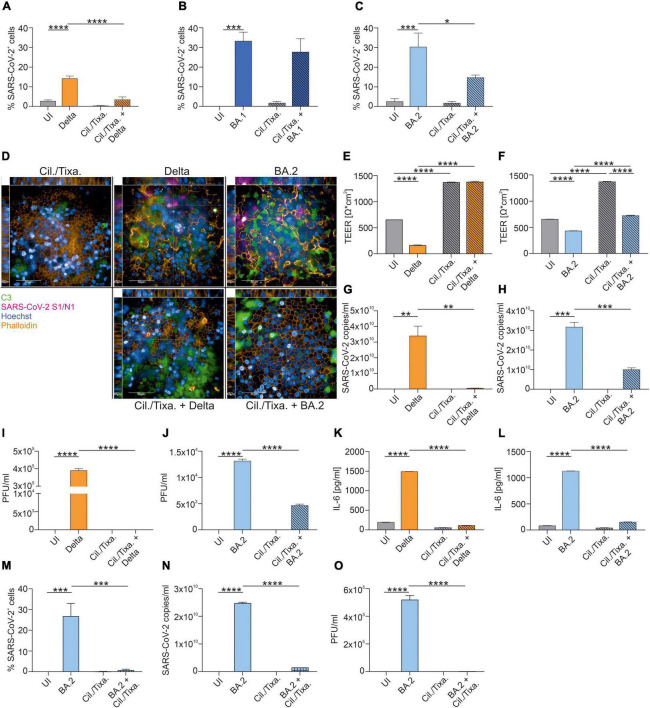
Efficacy of the prophylactic and therapeutic use of mAb cocktail Cilgavimab/Tixagevimab against SARS-CoV-2 Delta, BA.1 or BA.2. After 72 h of infection, NHBE cells grown on transwell filters were analyzed by immunofluorescence with the Harmony Software and percentage of SARS-CoV-2-positive cells were determined for Delta- **(A)**, BA.1- **(B)** and BA.2-infected cells **(C)**. Background signals due to the analysis were detected in UI and Cilgavimab/Tixagevimab (Cil./Tixa.) treated cells. **(D)** Representative pictures of XYZ stack are shown for the conditions where Cilgavimab/Tixagevimab pretreatment resulted in a significant reduction of SARS-CoV-2-positive cells shown in **(A–C)**. Scale bars represent 50 and 10 μm as indicated. After 72 h of infection, TEER was measured from Delta- **(E)** or BA.2-infected HAE ± Cilgavimab/Tixagevimab **(F)**. Viral RNA was analyzed in Delta- **(G)** and BA.2-infected cells ± Cilgavimab/Tixagevimab **(H)**. Plaque Assays of supernatants from Delta- **(I)** and BA.2-infected cultures ± Cilgavimab/Tixagevimab **(J)** were performed on Vero/TMPRSS2 cells. Concentration of IL-6 in the subnatants from Delta- **(K)** and BA.2-infected cultures ± Cilgavimab/Tixagevimab **(L)** were measured by ELISA. Cilgavimab/Tixagevimab was added to BA.2-infected cells after 6 h of infection and after 72 h of infection percentage of SARS-CoV-2-positive cells were determined **(M)**. Viral RNA was analyzed in BA.2-infected cells ± post-infected treatment of Cilgavimab/Tixagevimab **(N)**. Plaque Assays of supernatants BA.2-infected cultures ± post-infected treatment of Cilgavimab/Tixagevimab **(O)** were performed on Vero/TMPRSS2 cells. Statistically significant differences were determined by one-way ANOVA with Tukey correction. All values are means ± SEM; *P < 0.05; **P < 0.01; ***P < 0.001; ****P < 0.0001. At least three independent experiments were performed.

Further, we analyzed the efficacy of the prophylactic mAb cocktail Cilgavimab/Tixagevimab to reduce the percentage of SARS-CoV-2-positive cells. Pretreatment of HAE cells with Cilgavimab/Tixagevimab significantly reduced Delta- (*P* < 0.0001) (Figure 2A) and BA.2-mediated infection (*P* = 0.024) (Figure 2C), while no effect was observed upon infection of bronchial epithelial cells with the BA.1 variant (Figure 2B and Supplementary Figure 2A). Representative XYZ analyses of Cilgavimab/Tixagevimab-treated Delta- and BA.2-infected cells are shown in Figure 2D. Cilgavimab/Tixagevimab-treated infected tissues also illustrated an increased C3 activation, underlining innate immune activation. Moreover, the tissue integrity also revealed that prophylactic Cilgavimab/Tixagevimab treatment was able to rescue the drop in Delta-infected cells (Figure 2E), while we observed a significant increase in the TEER values in Cilgavimab/Tixagevimab-treated BA.2-infected cells (Figure 2F), which was still below the Cilgavimab/Tixagevimab control. Cilgavimab/Tixagevimab pretreatment almost completely prevented the detection of viral particles (Figure 2G) and significantly reduced viral titers, as analyzed by plaque assay, to 0 PFU/ml in Delta-infected cells (Figure 2I). In BA.2-infected cells, Cilgavimab/Tixagevimab prophylaxis significantly reduced SARS-CoV-2 copy numbers (Figure 2H) and viral titers (Figure 2J). The analysis of IL-6 concentrations revealed that Cilgavimab/Tixagevimab pretreatment resulted in a significant decrease of IL-6 levels in Delta- (Figure 2K) and BA.2-infected cells (Figure 2L). As the prophylactic use of Cilgavimab/Tixagevimab was the only treatment, which resulted in reduced infection with the Omicron BA.2 variant, we also analyzed the ability of this mAB combination to reduce an existing BA.2 infection. To mimic this scenario we added Cilgavimab/Tixagevimab 6 h after infection. We analyzed and quantified the percentage of SARS-CoV-2-positive cells in BA.2-infected cells in absence/presence of Cilgavimab/Tixagevimab (Figure 2M and Supplementary Figure 2B). In BA.2-infected HAE cells the therapeutic use of Cilgavimab/Tixagevimab resulted in a significant decrease of SARS-CoV-2-positive cells. Further Cilgavimab/Tixagevimab treatment of BA.2-infected cells almost completely prevented the detection of viral particles (Figure 2N) and significantly reduced viral titers, as analyzed by plaque assay (Figure 2O).

## Discussion

Here we studied the efficacy of a single dose of mAbs as therapy as well as prophylaxis in HAE during an infection with clinical isolates of SARS-CoV-2 Delta, BA.1 or BA.2. We report that the mAb Regdanvimab showed neutralization capacity against Delta, but lost antiviral activity against Omicron BA.1 and BA.2, which is in line with recent publications (1, 4, 11, 12). Sotrovimab treatment retained activity against BA.1 but was inactive against Delta and BA.2, which agrees with reported results from neutralization assays (1, 4, 11–14). The effectivity of Sotrovimab administration in BA.1-infected patients was also shown in a prospective study (15). Our data further highlight the reduced activity of Sotrovimab to treat BA.1 infection compared to Regdanvimab activity against Delta. Reduced efficacy of Sotrovimab to lower viral burden was also observed between BA.1 and the historical WA1/2020 D614G strain in an *in vivo* mouse model (16). In agreement with our study the authors also demonstrated that the post-virus inoculation of Sotrovimab did not diminish the viral RNA levels in BA.2-infected mice. Both, Regdanvimab and Sotrovimab did not exert any antiviral activity against Omicron BA.2, as shown previously using neutralization assays (2, 17, 18).

The prophylactic use of Cilgavimab/Tixagevimab raised hope to protect high-risk individuals with a poor antibody response after vaccination, a massively reduced immune function, or people, who cannot be vaccinated. We demonstrated that the prophylactic use of Cilgavimab/Tixagevimab nearly completely blocked subsequent infection with the Delta variant, while we were not able to observe any effect against the BA.1 VOC, which is in line with published neutralization assays showing reduced or no effect of Cilgavimab/Tixagevimab to neutralize BA.1 infection (2, 4, 11–13, 17). Most importantly, our data using an ALI 3D model of primary HAE cells demonstrate that only Cilgavimab/Tixagevimab significantly reduced infection with the Omicron BA.2 variant, which is in agreement with reports of neutralization assays using cell lines (2, 17, 18). To analyze the efficacy of Cilgavimab/Tixagevimab treatment to neutralize BA.2 infection, we used the approved prophylactic use and the therapeutic administration, which is not yet permitted by international drug agencies but showed success to treat SARS-CoV-2 infection in a non-human primate model (19).

Although we used physiological concentrations of the mAbs (8, 9), possible limitations of our study could be the use of one HAE donor as well as single concentrations for all tested mAbs. However, we and others could previously demonstrate that these early passages of HAE cells reduce donor-specific variations (6).

In summary, due to the absence of authorized therapeutic mAbs effective against the BA.2 variant, our findings emphasize the preventive use of Cilgavimab/Tixagevimab for high-risk individuals. The therapeutic administration of Cilgavimab/Tixagevimab, which is not yet approved by international drug agencies, even showed a stronger reduction of an infection with the BA.2 VOC compared to the prophylactic use of it and thereby emphasize the approval as therapeutic mAb-combination. Further, these data highlight the need of newly developed or adapted mAbs, which provide activity against current and future VOC.

## Data availability statement

The original contributions presented in this study are included in the article/Supplementary material, further inquiries can be directed to the corresponding author/s.

## Ethics statement

The studies involving human participants were reviewed and approved by the Ethics Committee of the Medical University of Innsbruck. The patients/participants provided their written informed consent to participate in this study.

## Author contributions

GW, CL-F, DW, and WP: conceptualization and resources. DW and WP: methodology and supervision. SD, DW, and WP: validation. SD, VZ, VK, HA, EL, LL, DW, and WP: formal analysis and investigation. SD, VZ, DW, and WP: data curation and writing—original draft preparation, and visualization. SD, VZ, VK, HA, EL, LL, GW, CL-F, DW, and WP: writing—review and editing. WP: project administration and funding acquisition. All authors have read and agreed to the published version of the manuscript.

## Supplementary material

Supplementary Figure 1 After 72h of infection, NHBE cells grown on transwell filters were analyzed by immunofluorescence with the Harmony Software. Representative pictures of XYZ stacks are shown for conditions where Regdanvimab or Sotrovimab treatment resulted in no significant reduction of SARS-CoV-2-positive cells illustrated in Figures 1A–C. Scale bars represent 50 μm and 10 μm as indicated.

Supplementary Figure 2 **(A)** Representative pictures of XYZ stacks are shown for conditions where Cilgavimab/Tixagevimab (Cil./Tixa.) pretreatment resulted in no significant reduction of SARS-CoV-2-positive cells depicted in Figures 2A–C. **(B)** Representative pictures of XYZ stacks are shown for BA.2-infected cells with therapeutic use of Cil./Tixa., which were analyzed for SARS-CoV-2-positive cells (depicted in Figure 2M).

## Abbreviations

VOC: variants of concern
RBD: receptor-binding domain
mAbs: monoclonal antibodies
EUA: Emergency Use Authorization
NHBE: normal human bronchial epithelial
ALI: air-liquid interphase
S1: spike 1
N: nucleocapsid
HAE: human airway epithelia
TEER: transepithelial electrical resistance.
