# Supplementary figures and images for: Cilgavimab/Tixagevimab as alternative therapeutic approach for BA.2 infections
Source: Front Med (Lausanne). 2022 Sep 29;9:1005589. doi: 10.3389/fmed.2022.1005589 (PMC9556863; doi:10.3389/fmed.2022.1005589)

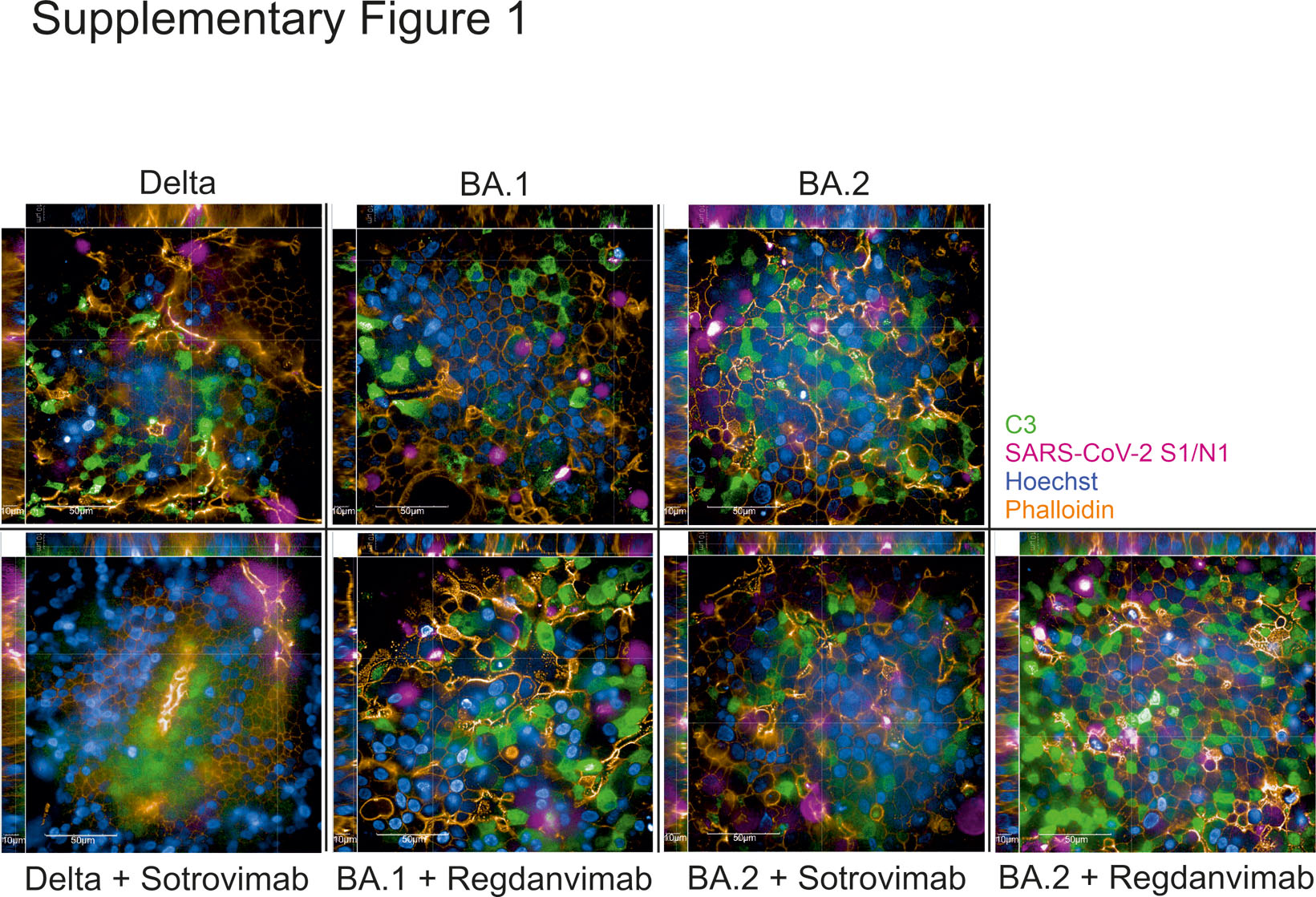

Supplement: Supplementary Figure 1 — After 72h of infection, NHBE cells grown on transwell filters were analyzed by immunofluorescence with the Harmony Software. Representative pictures of XYZ stacks are shown for conditions where Regdanvimab or Sotrovimab treatment resulted in no significant reduction of SARS-CoV-2-positive cells illustrated in Figures 1A–C. Scale bars represent 50 μm and 10 μm as indicated. [file Image_1.jpg]

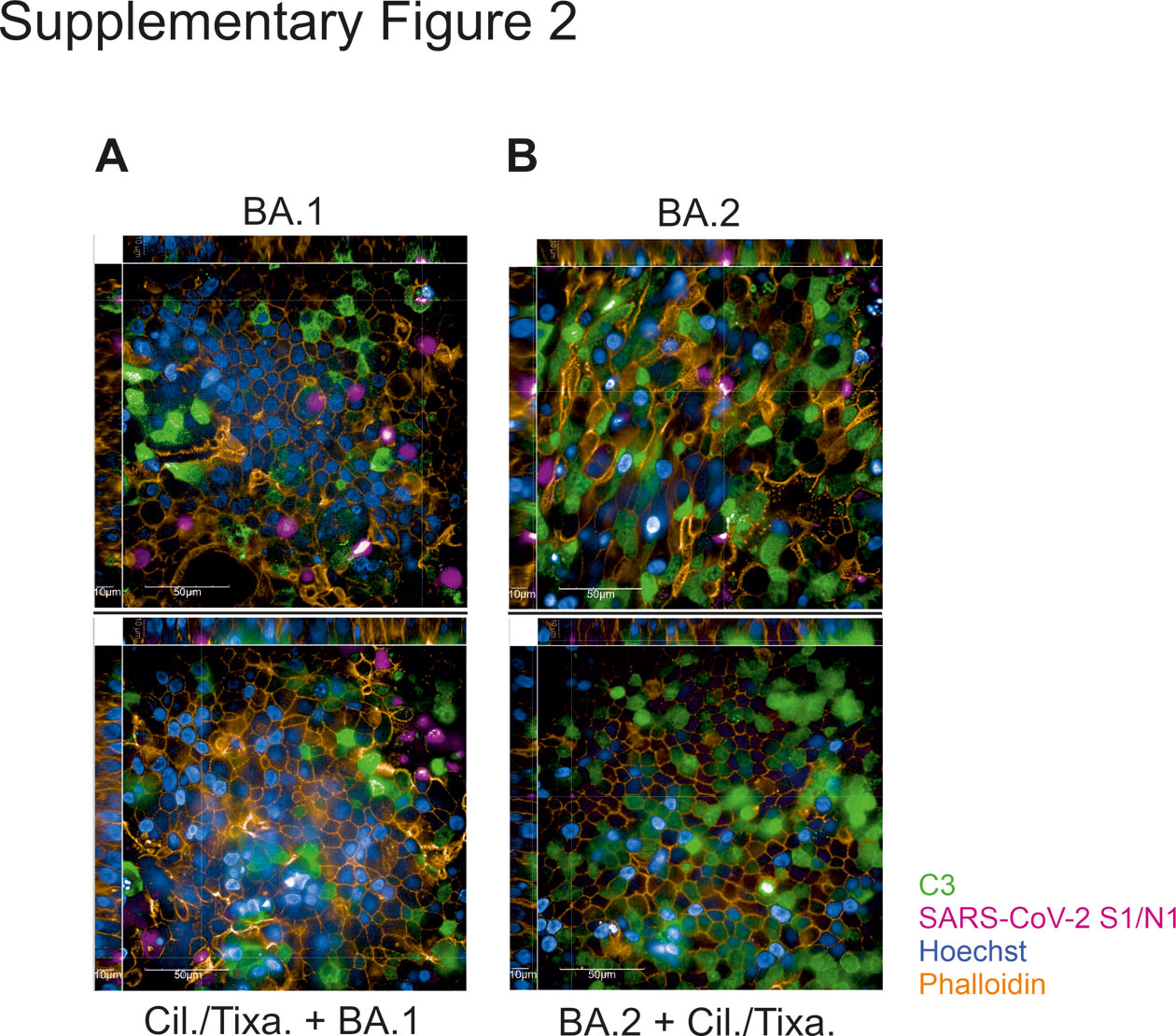

Supplement: Supplementary Figure 2 — (A) Representative pictures of XYZ stacks are shown for conditions where Cilgavimab/Tixagevimab (Cil./Tixa.) pretreatment resulted in no significant reduction of SARS-CoV-2-positive cells depicted in Figures 2A–C. (B) Representative pictures of XYZ stacks are shown for BA.2-infected cells with therapeutic use of Cil./Tixa., which were analyzed for SARS-CoV-2-positive cells (depicted in Figure 2M). [file Image_2.jpg]
